# Motor Vehicle Collisions during Adolescence: The Role of Alexithymic Traits and Defense Strategies

**DOI:** 10.3390/bs11060079

**Published:** 2021-05-21

**Authors:** Silvia Cimino, Eleonora Marzilli, Michela Erriu, Paola Carbone, Elisa Casini, Luca Cerniglia

**Affiliations:** 1 Department of Dynamic and Clinical Psychology, University of Rome, Sapienza, 00186 Rome, Italy; silvia.cimino@uniroma1.it (S.C.); eleonora.marzilli@uniroma1.it (E.M.); michela.erriu@uniroma1.it (M.E.); paola.carbone@uniroma1.it (P.C.); elisa.casini@uniroma1.it (E.C.); 2 Faculty of Psychology, International Telematic University Uninettuno, 00186 Rome, Italy

**Keywords:** motor vehicle collision, alexithymia, defense strategies use, adolescence

## Abstract

International literature has shown that adolescents represent the population most at risk of fatal and nonfatal motor vehicle collisions (MVCs). Adolescents’ alexithymic traits and significant use of immature defense strategies have been seen to play a key role. This study aimed to investigate the possible mediation role played by defense strategies use in the relationship between alexithymia and MVCs. Our sample consisted of 297 adolescents divided into four subgroups, based on the number of visits to the emergency department due to an MVC. We assessed adolescents’ alexithymic traits and defense strategies use through self-report instruments. Results showed that males reported a higher rate of MVCs than females. Higher rates of MVCs are associated with more alexithymic traits and maladaptive defense strategies use. Adolescents’ Acting Out and Omnipotence use significantly mediated the relationship between alexithymia and MVCs. Our findings suggest the recidivism of MVCs as an attempt to cope with emotional difficulties, with important clinical implications.

## 1. Introduction

Injuries among adolescents represent a research issue that has increasingly involved the scientific community [1,2,3]. In this field, motor vehicle collisions (MVCs) represent one of the main causes of death among 14- to 19-year-olds worldwide [4,5,6], who constitute the population most at risk even of nonfatal MVCs. Epidemiological studies on adolescents’ road collisions have reported a rate of prevalence of approximately of 50% [7,8,9], with important consequences for their psychological and physical well-being [10,11,12]. Most studies have shown a higher prevalence of injuries and road accidents among males [13,14,15,16,17], and results concerning females in relation to road accidents are few and inconsistent [18,19].

From a developmental point of view, adolescence represents a crucial phase of the human life cycle [20,21], due to irreversible changes in affective, cognitive, behavioral and social functioning [22,23,24], as well as the redefinition of relationships with family and peer groups [25,26]. Evidence from neuroscience studies has also shown the presence of important brain transformations during adolescence, such as a decrease in synapses and modifications in the limbic system [27,28,29]. These characteristics seem to be crucial in terms of adolescents’ emotional regulation and behavioral conducts [30,31,32], predisposing youths to greater susceptibility to different risky behaviors [33,34,35], including MVCs [36,37,38,39].

### 1.1. Literature Review

Adolescents’ MVCs represents a major public policy and health concern due to their severe psychological and physical consequences. Given the clinical relevance of the phenomenon, a better understanding of the underpinning mechanisms associated with multiple MVCs in adolescence is needed. In this context, some authors have considered MVCs as a consequence of adolescents’ practical inexperience and related errors (including loss of control, inadequate surveillance, speeding, and slippery roads) [40,41], distractions [42], deliberate violations of driving rules, or other factors commonly related to substance use [43,44]. However, a growing body of research has shown that MVCs during adolescence tend to occur many times over a few years [45,46], suggesting a more complex underlying etiology [47,48,49]. Interestingly, it has been recently suggested that personal factors (e.g., low education level and income, impulsivity, sensation-seeking, and low levels of altruism) [50,51], as well as their interaction with environmental/situational features (e.g., time delay, volume and traffic conditions) may lead to a higher risk of road collisions [52,53]. Nevertheless, to date, most of studies have focused on the possible consequences of adolescents’ MVCs (such as psychological and physical acute, chronic, fatal outcomes) [54,55,56]. Only a few studies have explored the possible individual vulnerabilities that may predispose the adolescent to a higher risk of MVCs [57,58,59,60]. In this field, clinicians and researchers rooted in the Developmental Psychopathology framework [61,62] have suggested that the presence of adolescents’ emotional difficulties preceding the accidents may lead to a higher risk of a MVC [63,64,65], as a form of acting out to cope with underlying psychological sufferance [66,67,68]. Some studies have focused on clinical samples of adolescents, evidencing significant associations between MVCs and ADHD [69], depression [70], personality disorders [71], and binge eating disorder [64]. However, the key role played by adolescents’ emotional difficulties has also been highlighted in non-clinical samples. Specifically, adolescents’ alexithymic traits and massive use of immature defense strategies have been widely prospectively associated with a broad range of externalizing problems [72], including behavioral addiction [73], substance use [74], and risk taking [75].

Interestingly, international literature has shown that the relationship between alexithymia and immature defense strategies is dynamic [76,77], evidencing a strong association between these variables [78,79], both in clinical [80,81] and non-clinical samples [82]. However, to date, research in the field of adolescents’ high-risk behaviors (such as MVCs) and externalizing difficulties has reported conflicting findings regarding the possible interplay between alexithymia and defense strategies use. Indeed, whereas some studies have suggested the mediation role of alexithymia in the relationship between dysfunctional defense mechanisms and adolescents’ high-risk activities [83], other studies have suggested that the relationship between alexithymic traits and maladaptive psychological functioning could be mediated by immature defense strategies use [76,79]. Research specifically focusing on MVCs in adolescence is scarce [45,46,84], but it has also shown significant associations both with alexithymia and immature defense strategies [63,64,85]. However, to date, no study has explored this complex relationship in relation to adolescents’ MVCs.

### 1.2. Current Study

A growing body of research in the field of high-risk behaviors during adolescence has underlined the key role played by adolescents’ emotional difficulties (i.e., alexithymia and massive use of maladaptive defense strategies) in increasing the risk of MVCs [45,46,75,84,85]. Consequently, it is important to implement knowledge of the complex relationship between these risk factors associated with adolescents’ MVCs to guide the planning of more effective prevention programs and reduce recidivism. Indeed, psycho-educational prevention programs aimed primarily at the promotion of adolescents’ copying skills related to safe driving, have produced mixed results for their effectiveness [86,87]. Based on these premises, we aimed to explore whether maladaptive defense strategies use may be a mechanism through which alexithymia affects the incidence of MVCs among adolescents.

Specifically, this study aimed to verify in a sample of male and female adolescents: (1) the possible differences between male and female youths in the recidivism of MVCs. Based on previous literature [13,14,15,16,17], we hypothesized a higher rate of MVCs among adolescent males. (2) The possible associations between MVCs, alexithymic traits, and maladaptive defense strategies use. We hypothesized that a higher rate of MVCs is associated with higher emotional difficulties in terms of alexithymia and significant use of immature defense strategies, in line with previous studies in the field [63,64,85]. (3) Possible mediation role played by adolescents’ maladaptive defense strategies use in the relationship between alexithymia and frequencies of MVCs. In accordance with recent studies that have shown that immature defense strategies use mediated the relationship between alexithymia and a wide range of psychopathological problems [76,79], we hypothesized that adolescents’ massive use of immature defense strategies may be a mechanism through which alexithymia leads to a higher incidence of MVCs.

## 2. Materials and Methods

### 2.1. Subjects, Recruitment and Procedure

Over a period of one year, we recruited *n* = 516 adolescents aged from 14 to 19 years through the collaboration of an Italian emergency department (ED) and private and public high schools in central Italy. To recruit adolescents who had experience with one or more MVCs, we used consecutive sampling, selecting all adolescents who accessed an ED over a period of one year. Given that it is a nonprobability sampling method, our sample should not be representative of the population due to inherent biases in the sampling process. Within the total sample, *n* = 285 of adolescents visited an ED for an MVC. From these subjects, as suggested by previous studies in this field [63,64,85], we excluded adolescents who were passengers at the moment of the collision (*n* = 29), adolescents with serious injuries (*n* = 20), adolescents who were positive for alcohol or drug use (*n* = 15), adolescents with a mental and/or physical disability (*n* = 21), adolescents who were following a psychological and/or psychiatric treatment (*n* = 17), adolescents who did not complete the assessment procedure (*n* = 13), and adolescents who did not consent to participate to the study (or whose parents denied consent to participate in the study) (*n* = 20). The final sample of adolescents, who had experienced one or more MVCs, consisted of *n* = 180 adolescents (73.3% males), with an average age of 16.62 (SD = 1.79). We also recruited a control group of *n* = 231 adolescents from which we excluded adolescents who had physical or mental disorders (*n* = 19), who were following psychological and/or psychiatric treatment (*n* = 21), who did not complete the assessment procedure (*n* = 35), and who refused to participate in the study (*n* = 29). The final normative sample (control group) consisted of *n* = 117 adolescents (50.4% males) aged from 14 to 19 years (M = 16.30, SD = 1.77).

**Figure 1:**
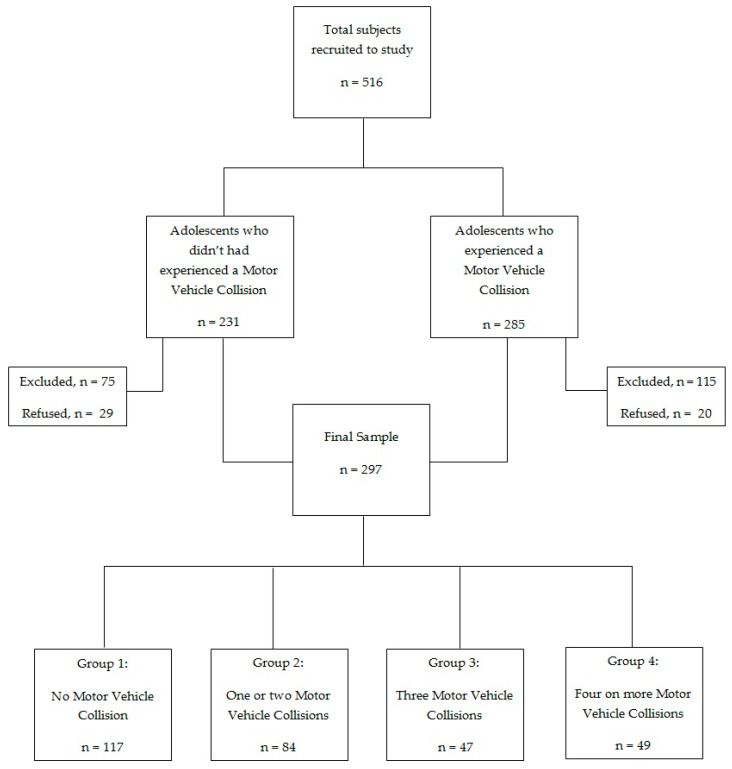
Recruitment process flowchart.

As suggested by Marcelli and colleagues [88], the sample (*n* = 297) was divided into the following four groups, based on the number of visits to the emergency department: Group 1 (*n* = 117): adolescents who had not experienced a MVC; Group 2 (*n* = 84): adolescents who had experienced one or two MVCs; Group 3 (*n* = 47): adolescents who had experienced three MVCs; Group 4 (*n* = 49): adolescents who had experienced four or more MVCs. The flowchart of the recruitment process is presented in Figure 1.

Most of the adolescents’ families were Caucasian (93.9%), and 66% had a household income between 28,001 and 55,000 euros per year. Of the adolescents, 78.11% were in intact family groups and 63% were firstborns. Before the start of the study, in line with the Declaration of Helsinki, La Sapienza University of Rome Ethical Committee approved the research plan. All the participants fill out an informed consent document. Finally, the privacy of the personal data was guaranteed. The complete description of the sample demographic characteristics is reported in Table 1.

All participants were administered an ad-hoc anamnestic questionnaire, evaluating different aspects (psychological, relational and social characteristics). The administration of questionnaires was made by expert psychologists inside a room made available, respectively, by an ED and private and public high schools. The following questionnaires were distributed to all study participants in a randomized order.

### 2.2. Measures

The Toronto Alexithymia Scale (TAS-20) is a self-report instrument for the assessment of alexithymia, composed of 20 items [89,90]. All items to be answered are scored on a 5-point Likert scale (from 1 = strongly disagree, to 5 = strongly agree). The scale is composed of three factors that reflect the more relevant elements of the construct of alexithymia. The first factor (Factor 1) assesses the ability to recognize emotions, differentiating them from physical sensations. Factor 2 refers to the ability to describe verbally one’s own emotions. Factor 3 assesses externally oriented thinking. The three-factor structure of the scale has been reported to be theoretically congruent with the alexithymia construct. Higher scores on the scales indicate difficulty in identifying and describing emotion and limited emotional functioning. Specifically, scores ≤50 indicate no alexithymia, scores between 51 and 60 are indicative of borderline alexithymia, and scores ≥61 indicate alexithymia. The TAS-20 showed good internal consistency and test–retest reliability (the total score’s internal reliability coefficient is 0.86). In the present study, Cronbach’s alpha for Factor 1 was 0.77, for Factor 2 was 0.79, and for Factor 3 was 0.87.

Adolescents’ use of defense strategies was measured by the Response Evaluation Measure (REM-71) [91,92]. The REM-71 is a self-report questionnaire composed of 71 items that assess various defense strategies, reporting the following: Acting out, Splitting, Displacement, Dissociation, Fantasy, Passive aggression, Projection, Repression, Omnipotence, Undoing, Conversion, Somatization, Withdrawal, Suppression, Denial, Humor, Intellectualization, Reaction Formation, Idealization, Altruism, and Sublimation. All items are scored on a 9-point scale from ‘‘strongly disagree” (scored as 1) to ‘‘strongly agree” (scored as 9). Higher scores on the scales indicate a massive and problematic use of the defense strategies. Research has shown good validity, reliability, and internal consistency for the scales of REM-71 [91]. In the present study, reliability of the REM-71 was also adequate (α = 0.74–0.88).

### 2.3. Statistical Analyses

Preliminary statistical analyses were conducted using descriptive statistics (frequencies, reliability of the measures, and mean scores). Chi-Square analysis was used to verify possible association between adolescents’ sex and MVCs. The number of cases in a cell can be considered significantly larger than those expected if the value of the relative adjusted residual is higher than 1.96 in absolute value. We reported information about the frequency distribution of the variables within a contingency table. Differences between the four groups in adolescents’ alexithymic traits and defense strategies use, considering the possible role played by adolescents’ sex, were examined using two-way multivariate analyses of variance (MANOVA). The differences between the sample means were identified used Bonferroni’s post hoc test. Finally, parallel mediation analyses were conducted to verify whether defense strategies use mediated the effect of alexithymia on the frequency of MVCs. We used Hayes’s PROCESS macro [93], considering adolescents’ sex and age as covariates. Before performing the mediation analyses, the scores of the independent variable and of the mediators were standardized. Indirect effects were evaluated with 95% bias-corrected confidence intervals (CI) based on 10.000 bootstrap samples. When CI do not include zero, it indicates that the effect is significant at α = 0.05. All analyses were performed using IBM SPSS software 25.0.

## 3. Results

### 3.1. Association between Adolescent’s Sex and Motor Vehicle Collisions

To verify the possible significant association between MVCs and adolescents’ sex, Chi-Square analysis was carried out. Results showed the presence of a significant association between adolescent sex and MVCs frequencies, χ^2^ (3, *n* = 297) = 20.75, *p* < 0.001. Specifically, as shown in Table 2, a detailed inspection of the contingency table showed that there was a significant association between female sex and the absence of MVCs, whereas male sex was associated with having had three or more MVCs in the past two years.

### 3.2. Alexithymic Traits and Defense Strategies Use in the Four Groups

To verify the possible differences between the four groups in alexithymic traits and defense strategies use, two-way MANOVA analyses were conducted. Results showed a non-significant interaction effect between sex and group, but there was a main significant effect of group [λ = 0.10, F = 11.66, *p* = <0.000, ηp^2^ = 0.53]. As shown in Table 3, analysis of the univariate effects showed the presence of significant differences between the four groups in the scores of Factor 1, Factor 3, and the Total score of TAS-20. Specifically, the Bonferroni post-hoc test showed that adolescents in Group 1 reported significantly lower scores of Factors 1 and 2 than other groups, whereas adolescents in Group 4 had the highest scores in the same dimensions of TAS-20. Moreover, they exceeded the clinical range cut-off in the Total score of TAS-20. At the same time, adolescents in Group 3 reported higher scores than adolescents in Group 2. However, both adolescents in Group 2 and Group 3 reported Total scores of TAS-20 in the borderline range. Moreover, there were significant group differences in the scores of Acting out, Omnipotence, Passive Aggression, Denial, Conversion, and Withdrawal. In particular, adolescents in Group 4 reported significantly higher scores of Acting Out, Omnipotence, and Passive Aggression than all other groups, and higher scores of Denial, Conversion, and Withdrawal with respect to adolescents in Groups 1 and 2. Adolescents in the control group (Group 1) reported significantly lower scores for all these defense strategies with respect to other groups, except for Conversion.

### 3.3. Adolescents’ Defense Strategies Use as Mediators of the Relationship between Alexithymia and MVCs

**Figure 2:**
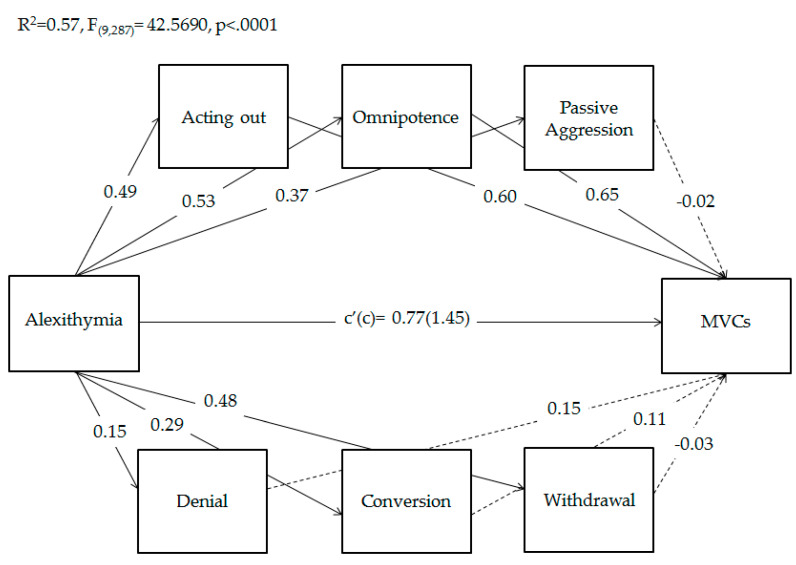
Parallel mediation of adolescents’ defense strategies use on the relationship between alexithymia and the frequency of motor vehicle collisions (MVCs). Coefficients shown are standardized path coefficients. Dotted lines represent non-significant parameters. c’ = direct effect; c = total effect.

Finally, based on previous results, to verify whether adolescents’ specific defense strategies use (i.e., Acting out, Omnipotence, Passive Aggression, Denial, Conversion, and Withdrawal) mediated the relationship between adolescents’ alexithymia and the frequency of MVCs, parallel mediation analyses were conducted using PROCESS macro [85]. As shown in Figure 2, results of mediation analyses showed that both the total and direct effects of alexithymia on MVCs were significant. Moreover, the direct effects of alexithymia on all considered defense strategies use were significant. In addition, adolescents’ use of Acting Out and Omnipotence significantly predicted MVCs, while the other defenses did not. Overall, this model explained 57% of the variance in adolescents’ MVCs.

Regarding indirect effects, Table 4 shows that the indirect paths via Acting out and Omnipotence were significant. Conversely, the single mediations of Passive Aggression, Denial, Conversion, and Withdrawal were not significant. Notably, the coefficient of direct effect was greater than that of indirect effects, indicating a partial mediation.

## 4. Discussion

The present study aimed to explore the complex relationship between adolescent’s MVCs, alexithymia, and the defense strategies use. We chose to explore the possible role played by adolescent psychological and emotional functioning because international literature has extensively highlighted that difficulties in recognizing and defining emotions and the significant use of immature defense strategies represent key risk factors in risk-taking behaviors among adolescence [75,94]. The same associations have also been found for adolescents who recurrently visited emergency departments due MVCs [63,64,85], but to our best knowledge, no study has considered the possible mediation role played by defense strategies use in the relationship between adolescents’ alexithymic traits and MVCs.

### 4.1. Main Findings

The first aim of this study was to verify sex-related differences in adolescents’ frequency of MVCs. Previous studies have reported conflicting results, with some studies finding a higher prevalence among adolescent males [4,14,15,16], but research reports few and inconsistent results concerning females and road accidents [95]. However, our study is in line with recent studies by Breen and colleagues [4] and by Le and colleagues [5], which evidence a greater risk of recurrent MVCs for adolescent males than females. Specifically, our findings showed that female sex is associated with the absence of MVCs, whereas there were significant associations between male sex and adolescent groups with a major number of MVCs (i.e., Groups 3 and 4). This could be due to the fact that adolescent males are more impulsive and tend to perceive behavior as less risky than females [96,97], with an overall greater tendency towards risk-taking [98,99,100]. In contrast, females tend to be more sensitive to the uncertainty and punishment potentially associated with risky behavior [101,102] and show a general aversion to risk compared with males [103]. Interestingly, a recent study by Cordellieri and colleagues [95] found the same level of risk perception during driving for males and females but significant sex differences in the level of concern about the risk of road accidents, with males being less concerned about risks and dangers. As suggested by some authors, this could be due the fact that adolescent males tend to overestimate their risky competence [104,105] and feel more invulnerable to driving risks compared with their female peers [106,107].

Then, we verified the possible differences in alexithymia and defense use between the four study groups. As expected, we found that adolescents’ maladaptive functioning, in terms of alexithymia and defense strategies used, was associated with a higher recurrence of MVCs. Specifically, adolescents with higher rates of MVCs reported greater difficulties in the ability to identify feelings and higher externally oriented thinking compared with adolescents of all other groups. Moreover, they exceeded the clinical range cut-off in the Total score of TAS-20. At the same time, they reported the highest use of immature defense strategies, including Acting Out, Omnipotence, Passive Aggression, Denial, Conversion, and Withdrawal. Our results are in accordance with previous studies that evidenced significant associations between adolescents’ tendencies to incur multiple MVCs, difficulties in identifying their feelings and emotions [64], and significant use of maladaptive defense strategies [63,108], suggesting that the accident could be placed in the area of non-integration between the adolescent’s affective life and cognitive abilities [67]. The relationship between alexithymia, immature defense strategies use, and unhealthy conduct has been widely investigated in the adolescent literature. In fact, adolescents with limited access to their emotions due to difficulties in identifying and coping with emotions are at higher risk of involvement in a wide range of negative risky behaviors, including substance use and abuse [109] and behavioral addiction [110,111]. In this field, clinicians and researchers from a psychodynamic perspective have considered risky behaviors (including nonfatal injuries) as an adolescent’s real attempt to cope with psychological discomfort [67,112]. Our findings provide further support to this evidence, suggesting that the recurrence of accidents may be considered a result of adolescents’ difficulties in managing their emotions and psychological sufferance [113,114,115]. At the same time, as suggested by Carbone [112], subsequent recurrent ED visits due to MVCs may be interpreted as an unconscious attempt to receive both psychical and psychological help from health operators.

### 4.2. The Mediation Role of Defense Strategies Use on the Relationship between Alexithymia and MVCs

Finally, our last aim was to verify whether the relationship between adolescents’ alexithymia and the frequency of MVCs could be mediated by maladaptive defense strategies use. Indeed, recent studies have evidenced that defense use mediated the effect of alexithymia and negative outcomes (such as psychological distress and psychopathological problems) [76,79], but this is the first study to explore this relationship in adolescents’ MVCs. Our results showed that both total and direct effects of alexithymia on MVCs were significant. Moreover, adolescents’ alexithymia significantly predicted all considered defense strategies use (i.e., Acting Out, Omnipotence, Passive Aggression, Denial, Conversion, and Withdrawal), in accordance with the study by Chung and colleagues [80] that found a predictive effect of alexithymia on maladaptive defense use. Moreover, adolescents’ use of Acting Out and Omnipotence significantly predicted MVCs. The same defense strategies partially mediated the relationship between alexithymia and MVCs. These findings supported the evidence that adolescents’ alexithymic traits represent an important risk factor for the recurrence of MVCs [64]. This influence seems to be exerted both directly and through the effects of adolescent massive use of Acting Out and Omnipotence. In this context, some authors posited that alexithymia may be considered as an individual primitive mental defense resulting as a strategy to reduce emotional involvement in a distressing situation [116,117]. A high presence of alexithymic traits has also been shown to further increase the use of other immature strategies [80,118], which transform individual emotional experiences [119]. Moreover, the incidence of high-risk behaviors during adolescence has been found to be associated with both alexithymia [120,121] and immature defense strategies use [83,122], which are in turn associated with each other [123]. Our study further supported this evidence, showing a specific role played by Acting Out and Omnipotence. In this field, according to Manciaux [124], self-punitive action is not deliberate in road accidents, but it would be considered as a distinct form of the dynamics of Acting Out: a dramatization in the external world of internal conflicts where psychic pain is replaced by a concrete and recognizable physical pain [109,110,111]. Other authors [125,126] have also highlighted the link between the incident and the need to increase the feeling of omnipotence; thus, in many cases, the recurrence of MVCs would express the adolescent’s attempt to deny their mortality and feel a sense of their invulnerability [66]. This dynamic has been suggested to be at the basis of a particular type of risk-taking behavior, defined by Marcelli [126] as ‘hordalic’, in which the escaped danger and the adrenalin rush would reinforce the feeling of omnipotence, even if fleetingly. However, the state of euphoria produced is only ephemeral and, like a drug, tends to induce addiction and trigger escalation towards increasingly extreme and risky behavior.

### 4.3. Limitations and Strengths

This study has some limitations. First, the cross-sectional nature of the design implies that the hypothesized causal links between alexithymia, defense strategies use, and MVCs must be treated with caution and should be verified by further longitudinal study. Moreover, the homogeneity of the sample limits the generalizability of the results to other races and geographical origins. Finally, we did not consider the possible role played by families and peer influence [127,128,129,130,131], which the literature has shown to play a key role in adolescents’ tendency to be involved in MVCs and other high-risk behaviors. Despite the above limitations, our study has several strengths. To our best knowledge, this is the first study to explore the possible interplay between adolescents’ MVCs, alexithymic traits, and immature defense strategies use. Our findings evidenced a key role played by alexithymia, which exerted its risk influence on adolescents’ MVCs both directly and indirectly via Acting Out and Omnipotence use, which may be informative for the planning of more targeted and effective intervention treatments.

### 4.4. Implications for Practice and Clinic Applications

Overall, our findings supported the importance of an early assessment of adolescents’ alexithymia and defense strategies use to prevent adolescents’ risk of MVCs. Generally, preventive programs on adolescents’ risk of MVCs are usually conducted in schools with the primary aim of incrementing teens’ copying skills related to safe driving. However, our results suggested that the planning of intervention strategies focused on the promotion of the ability to recognize and discriminate one’s own and others’ emotions is needed and may be more effective. Moreover, this study further supports the emerging evidence that adolescents who recurrently visited ED due to MVCs are seeking psychological help for their emotional difficulties. This could suggest the importance of an early assessment and the planning of secondary prevention programs directly in the ED, where adolescents could be psychologically supported in the identification of their psychological sufferance related to the MVC, to reduce recidivism, with important clinical, health and economic implications.

## 5. Conclusions

The recent literature in the field of adolescents’ risk taking has underlined that alexithymic traits and the significant use of maladaptive defense strategies represent crucial risk factors for the incidence of adolescents’ MVCs. Previous studies on the complex relationship between these two variables have reported mixed results. To date, no study has yet explored this relationship among adolescents who recurrently access an ED due to MVCs, suggesting the importance of implementing knowledge of these mechanisms to make the planning of preventive programs more targeted. This study has shown that adolescents’ alexithymia represents a key risk factor for MVCs, which leads to a higher risk of a MVC both directly that indirectly through adolescents’ Acting Out and Omnipotence use. Overall, these findings further supported the emerging evidence considering immature defense strategies use as a mechanism through which alexithymia affects the incidence of high-risk behaviors among adolescents, including MVCs, with important clinical implications.

## Figures and Tables

**Table 1:** Sample Demographic Characteristics.

	Group 1 (*n* = 117)	Group 2 (*n* = 84)	Group 3 (*n* = 47)	Group 4 (*n* = 49)
Age in years, M(SD)	16.30(1.77)	16.01(1.63)	17.28(1.75)	17.02(1.80)
Sex, *n*(%)				
Male	59(50.4)	55(65.5)	39(83.8)	38(77.6)
Female	58(49.6)	29(34.5)	8(17)	11(22.4)
Race, *n*(%)				
Caucasian	115(98.3)	76(90.5)	44(93.6)	44(89.8)
Other	2(1.7)	8(9.5)	3(6.4)	5(10.2)
Household income (EUR/year)				
0–15,000	12(10.2)	14(16.6)	5(10.6)	4(8)
15,001–28,000	8(6.8)	6(7.1)	4(8.6)	28(4)
28,001–55,000	75(64.1)	47(55.9)	35(74.4)	39(79.6)
55,001–75,000	19(16.2)	15(17.8)	3(6.4)	4(8)
>75,000	3(2.5)	2(2.3)	0	0
Family structure, *n*(%)				
Intact	84(71.8)	63(75)	41(87.2)	44(89.8)
Broken	33(28.2)	21(25)	6(12.8)	5(10.2)
Number of siblings, *n*(%)				
0	23(19.5)	31(37)	16(3)	21(42.8)
1–2	68(58.1)	37(44)	19(40.4)	15(30.6)
3–5	21(17.9)	15(17.8)	12(24.3)	13(26.6)
>5	5(4.2)	1(1.2)	0	0
Birth order, *n*(%)				
First position	102(87.1)	39(46.4)	27(57.4)	19(38.8)
Other positions	15(12.8)	45(53.6)	20(42.6)	30(61.2)

Group 1 = Adolescents who had not experienced a motor vehicle collision; Group 2 = Adolescents who have experienced one or two motor vehicle collisions; Group 3 = Adolescents who have experienced three motor vehicle collisions; Group 4 = Adolescents who have experienced four or more motor vehicle collisions.

**Table 2:** Association between adolescents’ sex and motor vehicle collisions (MVCs).

		Adolescent’s Group				Total
Sex		Group 1	Group 2	Group 3	Group 4	
Male	*n*	59	55	39	38	191
	Exp. Val.	75.2	54.0	30.2	31.5	
	% within sex	30.9%	28.8%	20.4%	19.9%	
	% within group	50.4%	65.5%	83.0%	77.6%	
	St. R	−4.0	0.30	2.9	2.1	
Female	*n*	58	29	8	11	106
	Exp. Val.	41.8	30.0	16.8	17.5	
	% within sex	54.7%	27.4%	7.5%	10.4%	
	% within group	49.6%	34.5%	17.0%	22.4%	
	St. R	4.0	−0.30	−2.9	−2.1	
Total	*n*	117	84	47	49	297

Exp. Val = Expected Values; % within sex = percentage within sex group; % within group = percentage within adolescent’s motor vehicle collision group; St. R = Standardized Adjusted Residuals. Group 1 = Adolescents who have not experienced a motor vehicle collision; Group 2 = Adolescents who have experienced one or two motor vehicle collisions; Group 3 = Adolescents who have experienced three motor vehicle collisions; Group 4 = Adolescents who have experienced four or more motor vehicle collisions.

**Table 3:** Univariate results of the differences between the three groups in youth’s family functioning, impulsivity, depression and anxiety problems between youth’s sex and IA.

		Adolescent’s Group					
		Group 1 M(SD)	Group 2 M(SD)	Group 3 M(SD)	Group 4 M(SD)	F_3293_	*p*-Value
**TAS-20**	Factor 1	7.12(1.72) ^a^	14.60(3.96) ^b^	17.44(4.37) ^c^	19.44(4.18) ^d^	**208.94**	<0.001
	Factor 2	15.81(5.37) ^a^	17.58(6.13) ^a^	16.31(4.98) ^a^	16.79(6.94) ^a^	1.56	0.19
	Factor 3	11.36(3.51) ^a^	20.86(4.57) ^b^	21.51(4.99) ^c^	25.46(5.65) ^d^	**152.86**	<0.001
	Total	34.30(7.27) ^a^	53.05(9.02) ^b^	55.27(7.78) ^b^	61.71(10.85) ^c^	**163.35**	<0.001
**REM-71**	Acting out	2.93(1.36) ^a^	4.28(1.41) ^b^	5.20(1.69) ^c^	6.28(1.92) ^d^	**62.93**	<0.001
	Splitting	6.08(1.70) ^a^	6.11(1.56) ^a^	5.79(1.86) ^a^	5.72(1.89) ^a^	0.83	0.47
	Displacement	4.31(2.02) ^a^	4.10(1.80) ^a^	4.34(1.86) ^a^	4.95(4.81) ^a^	1.10	0.34
	Dissociation	4.16(1.83) ^a^	4.42(1.77) ^a^	4.21(1.54) ^a^	3.90(1.88) ^a^	0.91	0.43
	Fantasy	4.05(2.06) ^a^	4.96(4.03) ^a^	4.55(2.11) ^a^	4.25(2.05) ^a^	1.84	0.14
	Omnipotence	3.45(1.26) ^a^	5.36(1.09) ^b^	5.56(1.64) ^c^	7.27(1.75) ^d^	**97.86**	<0.001
	PassiveAggression	4.20(1.74) ^a^	5.13(1.88) ^b^	5.08(1.73) ^c^	6.46(1.71) ^d^	**18.96**	<0.001
	Projection	3.08(1.80) ^a^	3.17(1.74) ^a^	3.16(1.91) ^a^	3.29(2.13) ^a^	0.15	0.92
	Suppression	4.18(1.85) ^a^	4.32(1.78) ^a^	4.35(1.89) ^a^	4.66(1.89) ^a^	0.79	0.49
	Undoing	5.13(1.85) ^a^	5.30(1.63) ^a^	4.84(1.92) ^a^	4.76(1.75) ^a^	1.29	0.27
	Sublimation	5.19(1.61) ^a^	5.20(1.55) ^a^	5.15(1.56) ^a^	5.34(1.68) ^a^	0.12	0.94
	Altruism	7.13(1.41) ^a^	7.13(1.44) ^a^	7.38(1.64) ^a^	6.75(1.83) ^a^	1.41	0.23
	Denial	4.39(1.94) ^a^	4.21(1.73) ^b^	5.43(1.46) ^b^	5.85(1.90) ^c^	**12.19**	<0.001
	Humor	5.07(1.71) ^a^	5.57(1.82) ^a^	5.35(1.56) ^a^	5.66(1.76) ^a^	1.97	0.11
	Idealization	6.11(1.74) ^a^	6.40(1.93) ^a^	6.35(1.96) ^a^	6.42(1.68) ^a^	0.54	0.65
	Intellectualization	5.42(1.55) ^a^	5.30(1.91) ^a^	4.89(1.56) ^a^	5.15(1.71) ^a^	1.19	0.31
	Reactive Formation	4.44(2.57) ^a^	4.48(1.84) ^a^	3.78(1.75) ^a^	4.14(1.64) ^a^	1.38	0.24
	Repression	4.92(1.60) ^a^	4.75(1.53) ^a^	4.83(1.48) ^a^	4.95(1.79) ^a^	0.24	0.86
	Conversion	1.33(.79) ^a^	1.61(1.11) ^a^	1.86(1.61) ^a^	2.25(2.06) ^b^	**6.18**	<0.001
	Somatization	3.42(1.85) ^a^	3.58(1.93) ^a^	3.63(1.80) ^a^	3.92(2.05) ^a^	0.80	0.49
	Withdrawal	3.76(2.24) ^a^	5.21(2.18) ^b^	5.86(1.85) ^b^	6.55(1.60) ^c^	**26.05**	<0.001

Different letters indicate significant differences. TAS-20 = Toronto Alexithymia Scale; Factor 1 = Difficulties identifying feelings; Factor 2 = Difficulties describing feelings; Factor 3 = Externally oriented thinking; REM-71 = Response Evaluation Measure-71; Group 1 = Adolescents who have not experienced a motor vehicle collision; Group 2 = Adolescents who have experienced one or two motor vehicle collisions; Group 3 = Adolescents who have experienced three motor vehicle collisions; Group 4 = Adolescents who have experienced four or more motor vehicle collisions. All bold values are statistically significant.

**Table 4:** Indirect effects of adolescents’ alexithymia on motor vehicle collisions through defense.

Indirect Effect	Effect(BootSE)	LLCI	ULCI
Alexithymia→Acting out→MVCs	0.29(0.05)	**0.16**	**0.46**
Alexithymia→Omnipotence→MVCs	0.34(0.08)	**0.19**	**0.52**
Alexithymia→Passive Aggression→MVCs	−0.01(0.05)	−0.12	0.09
Alexithymia→Denial→MVCs	0.02(0.01)	−0.01	0.06
Alexithymia→Conversion→MVCs	0.03(0.04)	−0.04	0.12
Alexithymia→ Withdrawal→MVCs	−0.01(0.06)	−0.15	0.09

MVCs = Motor Vehicle Collisions; BootSE = Boot-strapped standard error; LLCI = Lower level confidence interval; ULCI = Upper level confidence interval; all bold values are statistically significant.

## Data Availability

The data presented in this study are openly available in FigShare at doi:10.6084/m9.figshare.14402444.
